# A comprehensive intervention package improves the linear growth of children under 2-years-old in rural Bangladesh: a community-based cluster randomized controlled trial

**DOI:** 10.1038/s41598-022-26269-w

**Published:** 2022-12-19

**Authors:** Gulshan Ara, Kazi Istiaque Sanin, Mansura Khanam, Md. Shafiqul Alam Sarker, Fahmida Tofail, Baitun Nahar, Imran Ahmed Chowdhury, Anika Bushra Boitchi, Sarah Gibson, Kaosar Afsana, Sufia Askari, Tahmeed Ahmed

**Affiliations:** 1 International Centre for Diarrhoeal Disease Research, Bangladesh (icddr,b), 68, Shaheed Tajuddin Ahmed Sarani, Mohakhali, Dhaka, 1212 Bangladesh; 2 BRAC, 75 Mohakhali, Dhaka, 1212 Bangladesh; 3 BRAC James P Grant School of Public Health, BRAC University, 68 Shahid Tajuddin Ahmed Sharani, Mohakhali, Dhaka, 1212 Bangladesh; 4 The Children’s Investment Fund Foundation, 7 Clifford Street, London, W1S 2FT UK

**Keywords:** Nutrition, Paediatrics, Public health

## Abstract

Approximately one-third of children under the age of five are stunted in developing countries and many of them are micronutrient-deficient. We designed a comprehensive intervention package including egg/milk-based snacks to improve linear growth and dietary diversity among 6 to 12-month-old children in rural Bangladesh. In this 1-year community-based cluster randomized controlled longitudinal experiment, 412 mother–infant pairs were randomly assigned to receive either monthly food vouchers (for eggs, milk, semolina, sugar, and oil) to prepare egg and milk-based snacks for their children, along with multiple micronutrient powder (MNP), counseling on child feeding and handwashing, or regular government health communication alone (control; n = 206, treatment; n = 206). The trial was conducted in 12 clusters (small administrative units of sub-district). The primary inclusion criteria were ultra-poor households with limited resources and having children under 2-years-old. The primary and secondary outcomes were differences in children's length gain and dietary diversity. The effect of intervention on child growth was examined using a mixed effect linear regression model. Mean weight and length of the children did not significantly differ between groups at baseline. Around 90% of the children in both groups were breastfed. After receiving intervention for 12 months, LAZ score increased by 0.37 (CI 0.24, 0.51, *p* < 0.001) and risk of stunting reduced by 73% (OR: 0.27, CI 0.13, 0.58, *p* = 0.001). This comprehensive intervention package improved the growth and dietary diversity of children in extremely poor Bangladeshi households. A scaling-up of this intervention in contexts with limited resources should be taken into consideration.

**Trial registration:** This trial registered retrospectively at ClinicalTrials.gov as NCT03641001, 21/8/2018.

## Introduction

Stunting or linear growth faltering is a widespread form of chronic malnutrition that affects over one-fourth of children under 5-years-old globally, with the highest rates observed in South Asia and sub-Saharan Africa^1^. Stunting has a range of negative effects on a child ’s health, including shorter adult height, impaired cognitive development, weakened immune system, higher risk of illness, lower educational attainment, and a shorter life expectancy than average^2–6^. Studies conducted in low-resource settings suggest that stunting between the ages of 12 and 36 months is strongly associated to hindered cognitive and academic performance during the middle phase of childhood^7^. Analyses of early growth patterns across Africa and Asia show stunting starts before birth and in the first 2 years of life, the length-for-age Z-scores (LAZ) of stunted children decline rapidly^8^. Thus, many interventions emphasize the first 1000 days of life, from conception until children's second birthday as a critical window of opportunity^9^. However, the majority of stunting occurs during the supplemental feeding period (6–23 months old)^10^. Children can only consume small amounts of food during this period of rapid growth due to their limited gastric capacity^11^; therefore, complementary foods must be nutrient-dense and safely prepared to support their optimal growth and neurodevelopment^12^.

Over half of the essential nutrients required by infants are contained in eggs, which may also support the immune system. Eggs are an excellent source of high-quality proteins, vitamins, and minerals^13^. A study conducted in Ecuador that focused on the complementary feeding period demonstrated that giving children one egg per day for 6 months increased their LAZ scores by 0·63 (CI 0·38–0.88)^14,^. Similar to this, children in Uganda who received two eggs daily showed larger gains in height and weight over the course of 6 months than those who received one or no eggs (*P* < 0·05)^15^. Cow's milk is a nutrient-dense food source, and consuming milk is associated with higher levels of the hormone insulin-like growth factor (IGF-1), which promotes linear growth of the children^16,17^. In developing countries, about half of all children under the age of two have vitamin or mineral deficiencies, including zinc, iron, and vitamin A^18^. The use of micronutrient powders (MNP) in the home or point-of-use fortification of complementary food (CF) has been recommended to prevent micronutrient deficiencies^19^. Home fortification with MNP reduced anaemia by 31% (six trials, RR: 0.69; 95% CI 0.60, 0.78) when compared to no treatment or a placebo and iron deficiency by 51% (four trials, RR: 0.49; 95% CI 0.35, 0.67) among infants and young children^20^.

Dietary diversity (DD) in low-income countries reflects the overall adequacy and quality of nutrients in the diet of children^21–23^. Intake of a variety of food items is a key element of diversified food^24^. A study showed that high dietary diversity was associated to 15-fold and 26-fold lower risks of stunting in children aged 6 to 11 and 12 to 23 months compared to those with low DD^25^. However, families living in poverty sometimes struggle to provide their children with enough food^26^. Even though they are the most important sources of protein for children, animal-source foods are the first to be abandoned when families are struggling economically^27^. In Bangladesh, only 34% of 6 to 23-month-old children receive a minimum acceptable diet, and the quantity, diversity and proportion of animal-source foods in CF are inadequate, particularly in rural households^28^. Inadequate WASH practices also increase the risk of parasitic and diarrheal infections in children who do not receive the appropriate care^29^. A meta-analysis of ten trials including 16,473 children found an association between WASH programs and higher pooled mean height-for-age-z scores (7776 in the intervention group and 8687 in the control group; SMD = 0.14, 95% CI = 0.09, 0.19)^30^.

An extensive program implemented in Bangladesh revealed that rigorous counseling by frontline health workers improved CF knowledge and practices but did not result in child growth^31^. Another CF intervention study in Bangladesh that intensively promoted eggs showed that among young children (under 2 years old) in the intensive intervention group, egg consumption increased from 18 to 48% compared with an increase from 19 to 31% among the non-intensive group^32^. However, since stunting is a multi-factorial problem, an intervention aimed at addressing various risk factors, including societal, household, environmental, and socioeconomic factors, is likely to have a greater impact on linear growth than dietary interventions alone^33^. Considering the poor quality of CF in Bangladeshi context^34,35^ and the availability and cultural acceptability of eggs and milk, we hypothesized that a comprehensive intervention package including regular consumption of egg and milk-based snack, home fortification of CF with MNP, behavioural change communication (BCC) on child feeding and WASH would improve the growth of children. Thus, we conducted a cluster-randomized, community-based, controlled trial to explore the ability of this integrated intervention package to improve linear growth and reduce the risk of stunting among children under 2-years-old in a rural area of Bangladesh.

## Methods

### Study design and participants

The rationale and protocol for this randomized controlled community-based cluster trial were published previously^35^. The study was carried out in Harirampur, a sub-district of Manikganj District in the Division of Dhaka, Bangladesh. The population of this area is 2,81,274 and the average literacy rate is 30.2%^36^. Most people who inhabit this sub-district are involved in business, specifically agribusiness, grocery, and the fish trade. This area is bisected by the largest river in the country, the Padma River. Some of the unions of this sub-district are located along the Padma River. This upazila is very disaster-prone. A significant portion of the area is completely submerged by annual flooding. In cooperation with Bangladesh Rural Advancement Committee (BRAC), Harirampur upazila was specifically chosen as the study region, since one of BRAC's initiatives focuses on disadvantaged households with insufficient resources.

### Sample size

We assumed that the average value of LAZ for the control group was − 1.8. The hypothesis was based on a theme that, there would be a significant improvement by a mean difference of 0.4 after the given intervention provided, i.e., the average LAZ would reach − 1.4 in the intervention group. A two-sided significance level of 0.05, power = 0.80, standard deviation of the LAZ score = 1.2, and design effect = 1.3 was considered. The calculated sample size using this information in the first arm was 184. In contrast, equal sample size was used, and the sampling ratio between the control and treatment groups was 1:1. Then, the total sample size was 368. After adjusting for the 10% attrition, the required sample size was 412.

### Randomization and masking

We randomly selected six of the thirteen unions in Harirampur sub-district as the treatment area; the remaining seven unions served as the control area. One union from the control area was severely affected by seasonal flooding at the time of intervention; therefore, we could only work in six control unions. An investigator not involved in this study performed the computer-based randomization process to allocate the unions to the treatment or control areas. All severely low-income households in the treatment and control zones with children aged 6 to 12-months-old were identified and listed through door-to-door screening visits, then 206 children were randomly chosen from the list (which served as the sampling frame) and enrolled to receive the intervention if they met the inclusion and exclusion criteria. The treatment and control areas were geographically adjacent and have similar population demographics, but are separated by multiple rivers or canals that acted as buffers. Children with a LAZ < − 3 SD were excluded. The field staffs of icddr,b was not aware of the allocation of the treatment and control unions during screening, enrolment, or baseline data collection. Nevertheless, the evaluation team might learn from the community about the intervention package that was given to the treatment group. The evaluation team from icddr,b and the team from BRAC that carried out the intervention were two separate teams that were not in contact with one another because they were affiliated to two different organizations. However, both teams were unaware of the primary outcome of the study during the entire intervention period. Study enrolment and baseline surveys were completed in November–December 2017 before the 12-month intervention and follow-up were launched.

### Components of the intervention package

#### Egg and milk-based snack

Aiming to increase dietary diversity through regular consumption of animal-source protein and considering factors like the cultural context and compliance with the code for breastmilk substitutes, icddr,b developed the snack recipes. Two semolina recipes incorporating egg: *suji firni* (semi-solid consistency ideal for 6–12-month-olds) and *suji halwa* (solid consistency, recommended for older children) were developed and tested^35^. The mothers in the treatment group received food vouchers from BRAC each month worth around US $13 from their Nutrition Workers, allowing the mothers to acquire the ingredients for the snacks (suji firni/suji halwa) without having to pay cash. The mothers and caregivers used the vouchers to collect oil, suji and sugar monthly and milk and eggs three times a month from selected vendors. The Nutrition Workers made at least three monthly visits to the mothers and children to provide counsel on IYCF, how to prepare and feed their kids suji firni/suji halwa, how to use the MNP and how to wash their hands^35^.

#### Micronutrient powders (MNP)

During the home visits, the Nutrition Workers distributed a 1-month supply of MNP to the mothers in the treatment unions (containing iron, vitamin A, vitamin C, folic acid, and zinc). They showed the mothers how to divide the child's main meal into two portions and add the micronutrient powder to each portion. The Nutrition Workers instructed the mothers to use one sachet per meal for one child and to feed their children the fortified food within 30 min after preparation to prevent the formation of a metallic taste^35^.

#### Water, sanitation and hygiene (WASH)

BRAC distributed a handwashing station (a portable water tank with a tap and bowl) and soap to each of the participating households in the treatment unions to promote handwashing during critical times. Specialized child-focused WASH, and BCC materials were developed and distributed to inform and educate the households about the potentially harmful effects of consuming poultry feces and the consequences of environmental enteropathy^35^.

#### Behaviour change communication (BCC)

The Nutrition Workers used existing, standardized BCC resources (child-feeding counselling packages comprising flip charts and film) to counsel the mothers and primary caregivers in the treatment unions about recommended child-feeding practices^35^. The Nutrition Workers also used a wide range of supplementary BCC materials that were specially designed in consideration of the objective of the intervention. Promotion of egg/milk-based snack recipes and WASH were the main topics of the counselling. The mothers and primary caregivers were counselled on age-appropriate CF, managing breastfeeding, and CF challenges. The control group was only exposed to the regular government health and nutrition services and existing sources of information on maternal and child nutrition, and WASH. To promote continued participation in the data collection process and follow-up over the course of 1 year, the control group was given some incentives e.g., a feeding bowl, spoon and small toys.

### Study outcomes

The primary outcome of this randomized controlled trial was an improvement in the nutritional status (LAZ-score) of children aged 6–23 months old after receiving the comprehensive intervention for 12 months. The secondary outcome was dietary diversity among children.

### Data collection

Trained data collectors of icddr,b were responsible for the baseline assessment, follow-up, and endpoint data collection in both treatment and control areas. They followed a standard operating procedure (SOP) for data collection and anthropometry guidelines to maintain the quality of data collection. The data collectors underwent a validation exercise in addition to receiving the training.

#### Anthropometry

The anthropometric measurements (weight and length) were taken from the participants monthly using protocols recognized worldwide^37,38^. The length of the children was measured to the nearest 1 mm precision with a portable infantometer (Seca 417 model, seca gmbh &co.kg, Hamburg, Germany), and weight was measured with a baby scale to the nearest 0.01 kg precision (Seca 354 model)^39^. Length and weight were measured three times.

#### Infant and young child feeding

Standardized questions on child feeding practices that had previously been used in Bangladesh Demographic Health Surveys (DHS)^40^ were utilized to record IYCF practices during the monthly data collection. The questionnaire contains questions about current breastfeeding status, current usage of additional liquids, semi-solid, solid foods, and frequency of consumption. Dietary information was gathered using a list-based recall method to obtain the data on foods and drinks consumed by the children in the previous 24 h. Core IYCF indicators, such as minimum meal frequency, minimum dietary diversity, and minimum acceptable diet, can be evaluated using these standardized data-collecting techniques^41^.

#### Child morbidity

Data on the history of illnesses such as diarrhea, dysentery (blood and/or mucus), fever and coughs, and ear infections (purulent discharge from the ears) were collected monthly using a 2-week recall method. These questions were derived from the standard DHS infant morbidity recall questions and expanded to ask about ear discharge^42^. The passage of three or more times loose or watery stools in the last 24 h was defined as diarrhea; whereas invasive diarrhoea was defined as blood in the stools, and persistent diarrhea was defined as a single episode lasting longer than 2 weeks^43^. Coughing coupled with fast or rapid breathing or trouble breathing, with or without a fever, was considered acute respiratory disease^39^.

#### Household food security

The Household Food Insecurity Access Scale (HFIAS) was used for measuring household food security^44^. The HFIAS reflects three universal domains of the experience of inadequate household-level food access: (1) anxiety about household food supply; (2) insufficient quality, which includes food variety and preferences; and (3) insufficient quantity of food supply, the amount consumed, and the physical consequences of insufficiency^45^.

#### WASH and sociodemographic information

Data on hygiene practices (e.g., handwashing during critical times, kitchen conditions, cooking and reheating, hand-washing facilities)^44^ were collected bi-monthly. Self-reported socio-demographic data on age, education, gender, marital status, employment status and children's and maternal background information were obtained during the enrolment phase.

### Adherence to intervention

To gather monthly compliance-related data, one designated member of staff from icddr,b visited all household in the treatment unions. Monthly data on food vouchers, purchasing rations from vendors, preparing nutrient-dense snacks, feeding and sharing snacks, storing rations, preserving the prepared snacks, and pilferage, etc. were collected using semi-structured questionnaires. The empty MNP sachets were also collected to measure compliance at the household level. The fidelity of program implementation was evaluated using a process evaluation that emphasized the operation, implementation, and service delivery of the programme. The process evaluation examined how the study participants were chosen, how the voucher system was implemented, how participants used the vouchers, how effectively the Nutrition Workers conducted the various BCC/awareness sessions, how satisfied the staff were with implementation of the programme, and whether there were any obstacles in implementation^39^.

### Ethical approval

The Ethical Review Committee of the icddr,b granted ethical approval for this study (PR-17083). Written informed consent was obtained from mother or primary female caregiver of every enrolled child. The study was conducted according to the guidelines described in the Helsinki Declaration for research involving human participants.

### Statistical analysis

The World Health Organization's (WHO) Child Growth Standards were used to determine children's length-for-age (LAZ) and weight-for-age (WAZ) Z-scores^46^. Children with a length-for-age Z-score more than two standard deviations below the WHO Child Growth Standards median were classified as stunted. In addition, the rates of complementary feeding practices indicators (minimum dietary diversity, minimum meal frequency and minimum acceptable diet rate) and mean dietary diversity score was measured. The handwashing practice score was determined as a composite score of the indicators of handwashing practices (self-reported handwashing at crucial times; use of flowing water, soap, ash, sand, or stored water; and hand drying). Similarly, a composite score was also generated for handwashing facilities based on direct observation (presence of soap; a designated place for washing hands with a hand-cleansing agent; water available at the time of inspection, etc.). Incidence of diarrheal disease, fevers/colds and coughs per 100 children and median duration of illness were calculated for the entire 12-month period.

All inferential analyses were based on an intention-to-treat basis. Unadjusted bivariate analyses were carried out to examine the association between outcomes and potential covariates using the independent Student's t-test for continuous variables and Pearson's goodness-of-fit chi-square for categorical data. Mixed effect multiple linear regression model with exchangeable co-relation to adjust the intra-class correlation was used to assess the association of the intervention with children's length, LAZ, weight, and WAZ at different time points. Mixed effect linear regression with exchangeable correlation to adjust the intra class co-relation was used to assess the independent effect of intervention on those indicators. Variables were included initially in the multivariable models if baseline group comparisons had a p-value  < 0.20 or if factors were considered to be important for child growth based on previous research. Similarly, mixed effect logistic regression model was used to assess the effect of intervention on childhood stunting following the same procedure. We used the "mixed" and "melogit" commands to run the final mixed-effects model. Using the Poisson regression model, incidence rate ratios for symptoms associated with childhood morbidity were determined. The number of episodes of illness throughout follow-up (0–12 months follow-up) was the dependent variable in the final multiple Poisson regression model, and the offset was the log follow-up times after the relevant variables, and unions were adjusted^47^. Stata software (version 13·1; StataCorp, College Station, TX, USA) was used for all statistical analysis.

## Results

**Figure 1:**
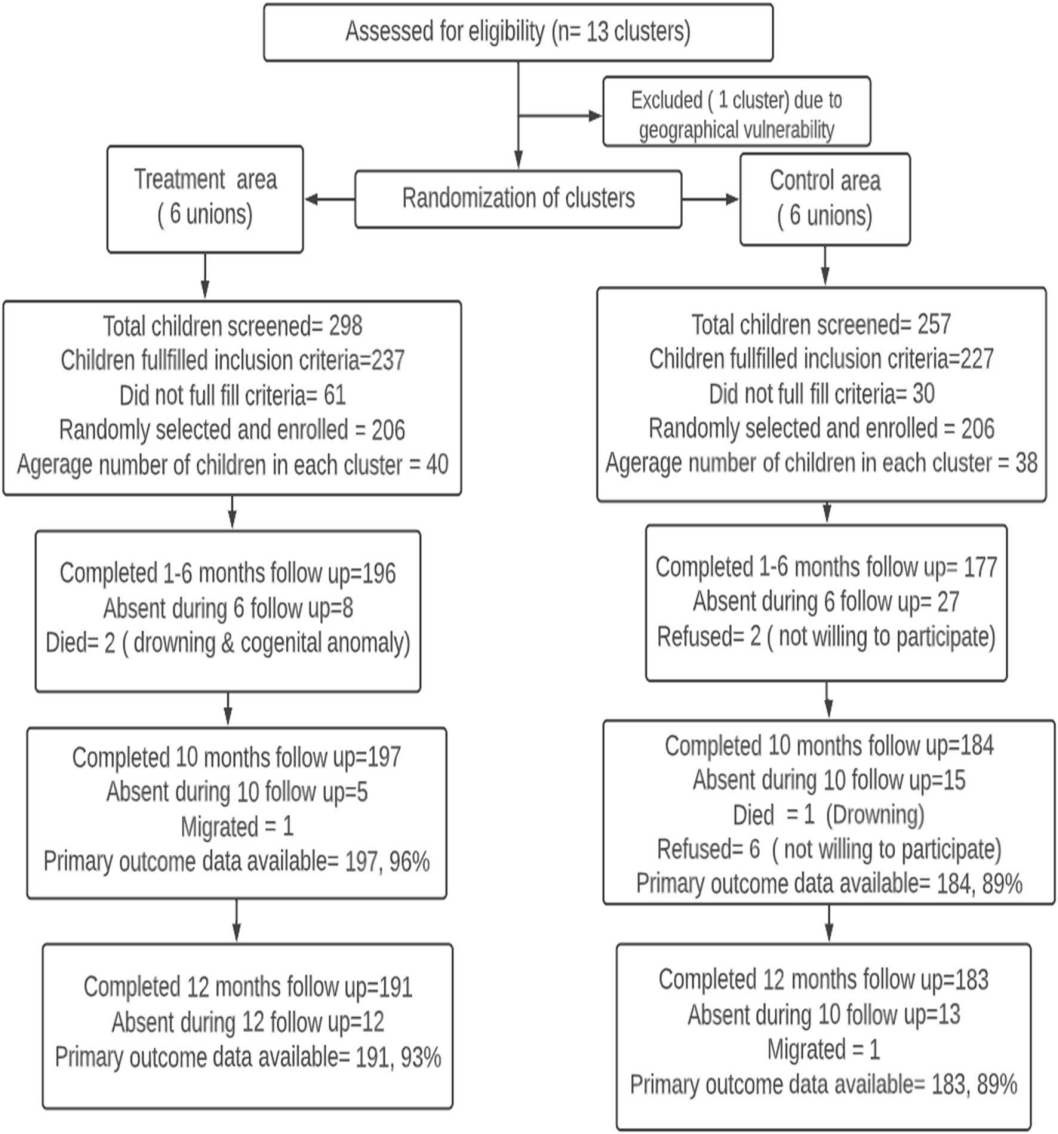
Consort diagram.

A total of 298 children from six treatment unions and 257 children from six control unions were screened; 237, and 227 children respectively fulfilled the inclusion criteria and 206 mother-children pairs were randomly selected for each group. Loss to follow-up was lower than anticipated. Six children in the treatment unions, and nine children in the control unions dropped out; key reasons included temporary reallocation, migration, and refusal to give data and/or anthropometric measurements every month. In the treatment unions, one child died of drowning, and another died of illness. In the control unions, one child died of drowning (Fig. 1).

The baseline demographic, maternal, and child characteristics are presented in Table 1. The average weight and age of the children at baseline were 8.2 kg and 9 months, respectively, for the treatment and control groups. The children in the treatment group had a higher average length (69.0 ± 3.0 cm) than the control group (68.4 ± 3.0 cm). However, mean LAZ did not differ significantly between groups at baseline (treatment: − 1.01 ± 0.89; control: − 1.07 ± 0.95). The majority of the children were breastfed. The mean age of the mothers was 25 years and around 56% of mothers in both groups had secondary or higher level of education; equal proportions (6.3%) of mothers in both groups were illiterate. The proportion of households with access to enough food was higher in the control group (67.0%) than in the treatment group (42.7%). The summary statistics for the children's average length and LAZ at baseline and end line by treatment and control clusters are shown in Table 2.

**Table 1:** Characteristics of the households and mother–child pairs in the treatment and control groups at enrolment.

	Treatment *n* = *206*	Control *n* = *206*
**Children**		
Age (month)	9.3 ± 1.8	9.0 ± 1.8
Girls	98 (47.6)	106 (51.5)
Boys	108 (52.4)	100 (48.5)
Length (cm)	69.0 ± 3.0	68.4 ± 3.0
Length-for-age Z-score	− 1.01 ± 0.89	− 1.07 ± 0.95
Stunting	27 (13.11)	30 (14.6)
Weight (kg),	8.23 ± 1.35	8.11 ± 1.19
Weight-for-age Z-score,	− 0.50 ± 1.14	− 0.47 ± 1.03
Still breastfed	187 (90.7)	182 (88.4)
Dietary diversity score	2.2 ± 1.4	2.1 ± 1.3
**Mothers**		
Age (years)	25.1 ± 4.9	25.0 ± 5.36
Height (cm)	151.2 ± 5.1	149.9 ± 11.3
Weight (kg)	50.3 ± 9.2	50.9 ± 10.3
BMI (kg/m^2^)	21.9 ± 3.6	22.9 ± 6.01
**Education**		
Illiterate	13 (6.3)	13 (6.3)
Primary or less	63 (30.6)	47(22.8)
Secondary or higher	117 (56.8)	116 (56.3)
College or higher	13 (6.3)	30 (14.6)
**Household**		
No of rooms used for sleeping	2.01 ± 0.94	2.21 ± 1.3
Household size	5.3 ± 1.7	5.6 ± 1.9
Household access to safe drinking water^a^	206 (100.0)	206 (100.0)
Use of improved toilets^b^	174 (84.5)	184 (89.3)
**Household food security status**		
Severely insecure	19 (9.2)	6 (2.9)
Moderately insecure	53 (25.7)	30 (14.6)
Mildly insecure	46 (22.3)	32 (15.5)
Secure	88 (42.7)	138 (67.0)

Values are presented as n (%) or mean ± SD.

^a^Safe drinking water: Sources by the nature of their construction that adequately protect water from outside contamination, in particular fecal matter.

^b^Improved toilet: sanitation facilities that hygienically separate human excreta from human contact.

**Table 2:** Summary statistics of major outcomes at cluster level.

Cluster levels	Baseline		End line	
	Length Mean (sd)	LAZ Mean (sd)	Length Mean (sd)	LAZ Mean (sd)
**Treatment clusters**				
1	69.68 (3.11)	− 0.81 (1.04)	84.32 (3.40)	− 0.45 (1.04)
2	68.01 (3.18)	− 1.21 (0.94)	81.98 (3.33)	− 1.02 (0.86)
3	68.93 (3.14)	− 0.97 (0.83)	83.48 (3.78)	− 0.64 (1.05)
4	69.63 (3.31)	− 0.87 (0.95)	84.30 (3.17)	− 0.38 (0.81)
5	69.81 (2.44)	− 0.86 (0.76)	84.12 (3.23)	− 0.45 (0.96)
6	68.29 (2.47)	− 1.37 (0.71)	82.54 (3.09)	− 0.88 (0.94)
**Control clusters**				
1	68.94 (2.95)	− 0.73 (0.93)	81.01 (3.29)	− 1.01 (0.97)
2	68.42 (3.32)	− 1.08 (0.91)	80.86 (2.74)	− 1.16 (0.67)
3	68.87 (2.91)	− 1.19 (1.02)	81.79 (3.50)	− 1.09 (1.11)
4	67.71 (3.51)	− 1.31 (0.83)	80.81 (3.04)	− 1.23 (0.65)
5	67.82 (2.99)	− 1.24 (1.11)	80.53 (3.50)	− 1.29 (1.03)
6	68.45 (2.61)	− 1.01 (0.84)	80.49 (3.05)	− 1.22 (0.94)

Values are presented as mean ± SD.

Table 3 shows the impact of the intervention on length and LAZ at various follow-up intervals for treatment and control group children. Children in the treatment group achieved a greater gain in length than the control group at 8th month (treatment: 78.79 and control: 77.17 cm) with a difference in length of 1.62 cm between the groups. This difference increased to 1.99 cm (treatment: 81.14, and control: 79.15 cm) and 2.62 cm (treatment: 83.52, and control: 80.89 cm) respectively, at the 10th and 12th months of intervention. From the 10th month of follow-up onward, the mean LAZ score was significantly higher in the treatment group than the control group (treatment: − 0.84, control: − 1.15). Following a year of intervention, the mean LAZ scores for the treatment and control groups were − 0.62 and − 1.16, respectively, over 12 follow-ups. The coef. of the LAZ score was 0.37 higher in the treatment group than the control group (CI 0.24, 0.51, p < 0.001) at 12th month. The risk of stunting decreased by 73% among the children in the treatment group compared to the control group at 12 months (adjusted OR: 0.27, CI 0.13, 0.58, p = 0.001), despite being 21% lower during the 10-month follow-up period.

**Table 3:** Effect of intervention on growth at different follow-up times.

	Treatment	Control	Unadjusted		Adjusted^c^		*ICC*
			Coef^a^/OR^b^ (95% CI)	*P-*value	Coef/OR (95% CI)	*P-*value	
**8-month follow-up (treatment n = 191, control n = 175)**							
Length (cm)	78.79 (3.43)	77.17 (3.14)	1.62 (0.7, 2.5)	0.001	0.95 (0.5, 1.4)	< 0.001	0.023
LAZ	− 1.01 (0.96)	− 1.17 (0.89)	0.16 (− 0.1, 0.4)	0.225	− 0.01(− 0.1, 0.1)	0.886	0.000
Weight (kg)	10.32 (1.75)	9.84 (1.41)	0.47 (0.1, 0.9)	0.023	0.53 (0.5,0.7)	< 0.001	0.000
WAZ	− 0.36 (1.27)	− 0.51 (1.10)	0.15 (− 0.1, 0.4)	0.290	0.41 (0.3, 0.6)	< 0.001	0.000
Stunting n (%)	30 (15.71)	30 (17.14)	0.90 (0.5, 1.6)	0.712	0.85 (0.5, 1.6)	0.624	0.008
**10-month follow-up (treatment n = 197, control n = 184)**							
Length (cm)	81.14 (3.53)	79.15 (3.14)	1.98 (1.1, 2.9)	< 0.001	1.42 (1.0, 1.9)	< 0.001	0.027
LAZ	− 0.84 (1.03)	− 1.15 (0.90)	0.32 (− 0.4, 0.7)	0.024	0.21 (0.1, 0.4)	0.003	0.000
Weight (kg)	10.72 (1.87)	10.25 (1.45)	0.47 (0.1, 0.9)	0.020	0.45 (0.2,0.7)	< 0.001	0.019
WAZ	− 0.36 (1.30)	− 0.52 (1.07)	0.15 (− 0.1, 0.4)	0.252	0.32 (0.1, 0.5)	0.001	0.017
Stunting n (%)	24 (12.18)	27 (14.67)	0.80 (0.4, 1.5)	0.490	0.79 (0.4, 1. 6)	0.498	0.012
**12-month follow-up (treatment n = 191, control n = 183)**							
Length (cm)	83.52 (3.40)	80.89 (3.19)	2.61 (1.9, 3.4)	< 0.001	2.05 (1.6, 2.5)	< 0.001	0.000
LAZ	− 0.62 (0.95)	− 1.16 (0.92)	0.54 (0.3, 0.8)	< 0.001	0.37 (0.2, 0.5)	< 0.001	0.000
Weight (kg)	11.17 (1.94)	10.57 (1.47)	0.60 (0.2, 1.0)	0.001	0.62 (0.4, 0.9)	< 0.001	0.000
WAZ	− 0.34 (1.32)	− 0.56 (1.09)	0.22 (− 0.03, 0.5)	0.083	0.44 (0.3, 0.6)	< 0.001	0.000
Stunting n (%)	12 (6.30)	33 (18.00)	0.30 (0.2, 0.6)	0.001	0.27 (0.1, 0.6)	0.001	0.000

Averages are presented as mean and standard deviation;

^a^In the first model co-efficient were estimated using mixed effect linear regression; unadjusted and adjusted analysis was accounted for clustering.

^b^Odds ratio were estimated using mixed effect logistic regression; unadjusted and adjusted analysis was adjusted for clusters.

^c^Adjusted for group, age, sex, child's baseline length, child's baseline weight, mother's height, and household asset index. Backward elimination stepwise covariate selection procedure was used; the models retained covariates with a P value of < 0.20 for the overall significance of the variables. No interaction terms with intervention were significant.

Effects of the intervention on dietary intake and handwashing and sanitation practices is presented in Table 4. At 4th and 8th month follow-ups, the children in the treatment group had consumed noticeably more meat, fish, and fleshy meals over the previous 24 h than the control group. Although the egg/milk-based snack was the major component of the intervention, we also assessed the intake of eggs and milk not included in the snack in last 24 h. Over 95% of children in the treatment group had consumed eggs in the previous 24 h at the 4th and 8th month follow-up, though egg intake slightly decreased to 91% in the treatment group at 12 months. Minimum dietary diversity and minimum acceptable diet were 25.2% and 28.5% higher, respectively, in the treatment group than in the control group at 12th month. Hand washing practice scores differed significantly between the treatment and control groups at 12th month; the facility score only differed significantly at 7th month*.*

**Table 4:** Effects of the intervention on dietary intake, handwashing and WASH practices.

	4-Month follow-up			8-Month follow-up			12-Month follow-up		
	Treatment	Control	*p*-value	Treatment	Control	*p*-value	Treatment	Control	*p*-value
**Children's dietary intake (yes/no)** n (%)									
Meat/fish intake	139 (70.2)	97 (55.4)	0.013*	156 (81.7)	123 (70.3)	0.027*	157 (82.7)	135 (73.7)	0.166
Egg intake	194 (97.9)	68(38.9)	< 0.001	188 (98.4)	104 (59.4)	< 0.001	175 (91.6)	95 (51.9)	< 0.001
Milk and milk product intake	194 (97.9)	116 (66·9)	< 0.001	191 (100.0)	144 (82.3)	< 0.001	186 (97.3)	104 (56.8)	< 0.001
Dietary diversity score (mean, SD)	5.7 (1.1)	4.13 (1.5)	< 0.001**	5.9 (0.89)	3·93 (1.3)	< 0.001**	5.86 (0.9)	4·24(1.2)	< 0.001**
Minimum dietary diversity n (%)	190 (95.9)	121(69.1)	< 0.001	191 (100.0)	121 (69.1)	< 0.001	187 (97.9)	133 (72.7)	< 0.001
Minimum meal frequency n (%)	191 (96.4)	153 (87.9)	0.008	191 (100.0)	167 (95.4)	< 0.001	188 (98.4)	162 (88.5)	0.001
Minimum acceptable diet n (%)	185 (93.4)	114 (65.5)	< 0.001	191 (100.0)	118 (67.4)	0.002	187 (97.9)	127 (69.4)	< 0.001

Handwashing and sanitation practices (mean, SD)	3-month follow up			7-month follow up			12-month follow up		
Handwashing practice score	8.2 (0.6)	8.1 (0.7)	0.549**	8.5 (0.04)	8.27 (0.6)	0.079**	8.32 (0.05)	8.20 (0.40)	0.050**
Handwashing facilities score	3.7 (0.0)	3.6 (0.4)	0.088	3.9 (0.02)	3.76 (0.4)	< 0.001	3.81 (0.60)	3.69 (0.75)	0.172

*p values were calculated from mixed effect logistic regression model where cluster were adjusted.

**p values were calculated from mixed effect linear regression model where clusters were adjusted.

### Childhood morbidities during the intervention

The incidence of fever per 100 child months was 25.7% and 23.2%, respectively, which was 2.5% higher in the treatment group than in the control group. Children receiving treatment had an 11% higher risk of fever than those in the control group (IRR: 1.11, 95%; *p* = 0.003). The median duration of cold, cough and runny nose was higher in the control group compared to the intervention group. Similarly, the incidence of colds/runny noses was 2% higher among the control group than the treatment group. The incidence of coughs and diarrheal disease were comparable between the groups (Table 5).

**Table 5:** Effects of the intervention on childhood morbidities over the entire 12 months.

	Treatment		Control		Unadjusted		Adjusted	
	Median (days) IQ^a^	Incidence rate per 100 child-months	Median (days) IQ	Incidence rate per 100 child-months				
		(95% CI)		(95% CI)	IRR^b^ (95% CI)	*p*-value	IRR (95% CI)	*p*-value
Fever	9 (6, 15)	25.7 (24.6, 27.0)	8 (5, 12)	23.3 (21.7, 24.9)	1.11 (1.0, 1.2)	0.011	1.11 (1.0, 1.2)	0.003
Cough	14 (8, 24)	28.4 (27.7, 29.1)	15 (9, 24)	27.9 (26.0, 29.8)	1.02 (1.0, 1.1)	0.588	1.04 (0.9, 1.2)	0.450
Cold &/runny nose	28 (19, 38)	50.1 (47.0, 53.4)	32 (21, 45)	52.0 (48.9, 55.4)	0.96 (0.9, 1.0)	0.361	0.97 (0.9, 1.1)	0.576
Diarrhea &/dysentery	5 (3, 8)	12.1 (11.0, 13.3)	6 (4,10)	11.8 (10.8, 13.0)	1.02 (0.9, 1.2)	0.729	1.01 (0.9, 1.2)	0.926

^a^Inter quartile range.

^b^IRR were estimated using Poisson regression with sandwich variance estimator at the cluster level. The dependent variable was number of episodes of illness during follow-up (0–12 months follow-up); the offset was the log total number of events during follow-up. Adjusted for age, sex, child's baseline length, mother's height, household asset index, and study group; the control group was treated as the reference.

## Discussion

Multiple elements, including dietary and environmental factors, influence children's growth. Therefore, it is unreasonable to anticipate that a single intervention will significantly improve the growth of children. Early childhood is a time of rapid growth and children require a diet rich in nutrients and quality sources of protein^48^. Eggs provide large quantities of essential macronutrients and have high digestible essential amino acid scores^49,50^. Moreover, a recent meta-analysis in the *Lancet* concluded that macro and micronutrient supplementation positively improved the LAZ and cognitive development scores among children in LMICs^51^. Thus, in order to increase the growth of children from low-income rural Bangladeshi households, this innovative community-based cluster RCT study examined the impact of daily provision of an egg- and milk-based snack through food vouchers, along with MNP supplementation, child nutrition counselling, and WASH. This is the first intervention to use food vouchers to improve the complementary feeding practices and in turn, the growth of young children from ultra-poor rural households. This comprehensive 1-year nutrition-specific and nutrition-sensitive intervention significantly improved the linear growth of children aged 6 to 24 months old by 2.62 cm, reduced the risk of stunting by 76%, and improved child feeding indicators and handwashing practices.

In an experimental supplementary food study in Ecuador, Iannotti and colleagues found that providing one egg per day for 6 months to 80 children aged 6–9 months increased the LAZ by 0.63 compared to control children^14^. However, another low-attrition, high-adherence RCT in rural Malawi did not find eggs increased length gain or reduced the prevalence of stunting^52^. The average age of the children in the Ecuadorian trial was 7 months; our participants were ~ 9-months-old^52^. The length velocity is normally 2.1 cm/month during the first year^53^, which could explain why the younger Ecuadorian children achieved greater gains in length within 6 months. Moreover, the Ecuadorian trial only lasted 6 months, whereas our trial was 12 months long. We anticipated that supplying eggs and milk for an entire year would be necessary to observe a beneficial effect on linear growth. Finally, compared to our study (control: − 1.07 vs. treatment: − 1.01), the Ecuadorian children had lower baseline LAZ scores (control: − 1.71, treatment: − 2.09) and the Malawian children had higher baseline LAZ scores (control: − 0.86 treatment: − 0.91). Mustafa and colleagues reported that provision of 1 egg and 150 mL milk (90 days) and MNP (1 RDA, 60 days) led to a better effect size among children aged 12‒18-months-old who were stunted (LAZ < − 2 SD) than those at risk of stunting (LAZ < 1 to − 2 SD), which suggests that the baseline LAZ significantly impacts the treatment effects^54^. Thus, the differences in the LAZ scores could also explain the difference in the effect sizes of our study and the Ecuadorian trial. Overall, compared to other published interventions, our intervention led to a greater reduction in the rate of stunting.

The current study found that, at 12 months, almost 95% of the children in the treatment group achieved minimum dietary diversity and minimum acceptable diet criteria, which was 25% higher than the control group. The provision of intensive IYCF counselling might have played a crucial role in the high adherence to regular consumption of the snacks. Moreover, semolina cooked with milk is a widely practiced complementary food in Bangladesh and eggs are widely available and acceptable^50^. Thus, this study demonstrates that the milk and egg-based snack intervention is pragmatic and feasible in the Bangladeshi context.

Children in developing countries frequently experience a variety of illnesses that limit their ability to absorb nutrients, reduce their overall nutrient intake, and cause them to lose more nutrients than they take in, which can impede linear growth. Using eggs and milk as a sustainable food-based strategy to address their macronutrient deficiencies has a huge potential to improve dietary quality because these foods are abundant in energy and an excellent source of high-quality protein^55^. When compared to the control group, the treatment group children throughout this intervention experienced more common childhood illnesses such fever, cough, colds/runny noses, and diarrhea. However, the intervention package did not significantly reduce common childhood morbidities. Although the treatment group exhibited a greater length increment than the control group, the incidence of fever was 2.5% higher among the children in the treatment group. The improved linear growth of the treatment group could possibly be due to the 12-month-long supplementation of animal source food combined with MNP. Nevertheless, the egg intervention in the Ecuadorian trial without MNP improved growth without reducing morbidity. Children frequently exhibit multiple micronutrient deficiencies accompanied by macronutrient deficiencies due to an insufficient food supply^56^. Hence, fortification and supplementation programs can help to prevent and treat micronutrient deficiencies^57^. The addition of animal sources of food in the diet, even in modest amounts, can help prevent different deficiencies since many supplementation and fortification programs alone may not address macronutrient deficiencies^58^. Therefore, the improved growth observed in the treatment group further suggests that our proposed egg- and milk-based intervention package combined with MNP supplementation improved children's health.

The nutritious snack recipe in this trial could be promoted through community-based nutrition intervention programmes. However, the snack cost might be a burden for low-income families because of the high price of eggs. For instance, cost was the major constraint to routine egg consumption in rural Zambia^59^. Egg is a costly source of calories in low-income countries, and the cost of the egg is strongly associated with the consumption of eggs by young children^50,60^. Given the relationship between socio-economic status and egg consumption, egg intake is likely to increase as household incomes rise^50^. Hence, efforts to increase availability and access to eggs are also needed in addition to improving household incomes.

It is important to acknowledge several limitations of this study. First, there were some baseline anthropometric differences between the two groups, despite the randomization process producing comparable groups in terms of all other observed features. However, the regression models were adjusted for the baseline anthropometric measures and clusters to account for these variations. Moreover, we could not assess the effects of the individual components or combinations of the intervention components. The high level of adherence to the intervention resulted from the BCC and regular home visits by BRAC personnel; hence, it can be difficult to generalize the implementation of this type of intensive intervention.

Moreover, evaluations of dietary sufficiency and iron and zinc deficiency are necessary to confirm the beneficial effects of the egg- and milk-based snack and micronutrient fortification. The lack of masking is another important limitation of this food-based community intervention. Finally, although this intervention seems expensive in low-resource contexts, such investment may be justified in terms of the yield in terms of human health and productivity. Therefore, this trial provides evidence for the development of an integrated intervention to improve the linear growth of disadvantaged young children.

## Data Availability

Data described in the manuscript, codebook, and analytic code can be made available upon a reasonable request. The Principal Investigator/ corresponding author will be responsible for providing the data (gulshan.ara@icddrb.org) if required.

## Abbreviations

ASF: Animal source food
brac: Bangladesh Rural Advancement Committee
CIFF: Children Investment Fund Foundation
coef: Coefficient
IGF-1: Insulin-like growth factor-1
IRR: Incidence risk ratio
IYCF: Infant and young child feeding
LAZ: Length-for-age Z-scores
LMIC: Low and middle income countries
MNP: Multiple micronutrient powder
RCT: Randomized controlled trial
RDA: Recommended dietary allowance
SD: Standard deviation
SOP: Standard operating procedure
WASH: Water, sanitation, and hygiene
WAZ: Weight-for-age Z-scores
WHO: World Health Organization
